# Symptom diaries as a digital tool to detect SARS-CoV-2 infections and differentiate between prevalent variants

**DOI:** 10.3389/fpubh.2022.1030939

**Published:** 2022-11-14

**Authors:** Barbara Grüne, Sabine Kugler, Sebastian Ginzel, Anna Wolff, Michael Buess, Annelene Kossow, Annika Küfer-Weiß, Stefan Rüping, Florian Neuhann

**Affiliations:** ^1^Health Department Cologne, Cologne, Germany; ^2^Department of Knowledge Discovery, Fraunhofer Institute for Intelligent Analysis and Information Systems (IAIS), Sankt Augustin, Germany; ^3^Institute for Hygiene, University Hospital Muenster, Muenster, Germany; ^4^Heidelberg Institute for Global Health Heidelberg University Hospital, Heidelberg, Germany; ^5^School of Medicine Lewy Mwanawasa Medical University, Lusaka, Zambia

**Keywords:** SARS-CoV-2, digital symptom diaries, prevalent virus variants, machine learning, classification, symptom combinations, health department

## Abstract

The COVID-19 pandemic and the high numbers of infected individuals pose major challenges for public health departments. To overcome these challenges, the health department in Cologne has developed a software called DiKoMa. This software offers the possibility to track contact and index persons, but also provides a digital symptom diary. In this work, the question of whether these can also be used for diagnostic purposes will be investigated. Machine learning makes it possible to identify infections based on early symptom profiles and to distinguish between the predominant dominant variants. Focusing on the occurrence of the symptoms in the first week, a decision tree is trained for the differentiation between contact and index persons and the prevailing dominant variants (Wildtype, Alpha, Delta, and Omicron). The model is evaluated, using sex- and age-stratified cross-validation and validated by symptom profiles of the first 6 days. The variants achieve an AUC-ROC from 0.89 for Omicron and 0.6 for Alpha. No significant differences are observed for the results of the validation set (Alpha 0.63 and Omicron 0.87). The evaluation of symptom combinations using artificial intelligence can determine the individual risk for the presence of a COVID-19 infection, allows assignment to virus variants, and can contribute to the management of epidemics and pandemics on a national and international level. It can help to reduce the number of specific tests in times of low labor capacity and could help to early identify new virus variants.

## Introduction

Innovative tools for notification, monitoring, and reporting are required due to the high rates of people infected with SARS-CoV-2 and their contacts in Germany. Positively tested persons and their close contacts, residing in Cologne, are managed using a specifically developed software DiKoMa (1). This digital tool, developed for COVID-19, provides symptom diaries that can either be used by health authority staff or self-administered by affected individuals. A dataset of around 90,000 patients with COVID-19 and 75,000 contact persons is collected from May 2020 until February 2022 from DiKoMa.

The symptomatology in the COVID-19 infections shows various multiple and often unspecific signs and symptoms that are also found in other infectious diseases. Rarely, a single symptom occurs alone in a COVID-19 infection, but symptom combinations, like cough, are accompanied by dysgeusia and dysosmia. Furthermore, COVID-19 is highly infectious, but the disease cannot be clearly delineated from other diseases and must be confirmed by a laboratory test. Having the same symptoms at the beginning of the disease, the course of the disease can highly vary in severity and additional symptoms. The condition can change on a short notice, and the clinicians must react quickly. This constitutes a challenge for the clinical diagnosis and the assessment of clinical severity.

Hence, in routine practice during the pandemic, diary entries are used in contact management to prioritize follow-up interventions such as follow-up phone calls or sending a mobile clinical team to identify and manage index cases with an increased risk for a severe course of the illness. By evaluating the records of contact persons, it is also possible to identify those who develop active disease and are referred for testing. To achieve this, daily defined database queries are performed using criteria such as age, existing co-morbidities, self-reported deterioration of general health status, and newly developed symptoms in contact persons. In times of high infection rates, there has often been too little laboratory capacity in relation to the number of necessary PCR tests and the period until the result of the PCR test was submitted increased to 4–5 days; thus, there was a need to detect the symptomatic contact persons, which were COVID-19-infected, as early as possible. In addition, alteration of the clinical symptoms suggested a new predominant variant, whereas the sequencing of PCR tests for new virus variants was challenging and not possible all the time. This led to the question of whether it is also possible to detect infections and virus variants based on symptom patterns.

Beyond this practical application, the data are used to train a machine learning (ML) model to recognize patterns in early symptom profiles (SP) in relation to the prevailing dominant variant. This evaluation shows the difference between typical symptom patterns of actual infected individuals and symptom patterns of their contact persons with a negative test result (rapid test or PCR test) as well as their relation to the prevailing dominant virus variant.

## Related work

Since the beginning of the COVID-19 pandemic, many apps and tools are developed to track symptoms, for prevention of an infection, for health information and education, or for home-based monitoring (2). Tracking and documentation of symptoms as well as the analyzation of the data offer opportunities for a better understanding of the disease course and severity and tailoring of containment measures. Many models are developed to predict a possible infection of patients only by the reported symptoms, as the review of Huang et al. shows (3). For example, Manni et al. (4) developed a logistic regression to distinguish between index and contact persons and Drew et al. (5) published an app which revealed symptom combinations which are predictive for a positive COVID-19 test. Spinato et al. (6) created and validated a differentiated questionnaire to identify index cases based on symptomatology.

Nevertheless, symptom diaries are used not only to distinguish between infection or non-infection, but also to determine symptom change during an infection (7), or the symptom intensity over the time course (8). The databases of these publications are mostly covering short time periods (e.g., a month) allowing only to investigate symptom diaries from patients with the prevailing dominant variant during this period. However, from a medical and public health perspective, not only the COVID-19 infection of a person is important, but also the variant of the virus, because of differences in contagiosity, clinical course, and severity. For the differentiation of variants, either clinical diagnostic tool such as CT or X-ray imaging (9) or molecular genomic sequencing (10) is used. Menni et al. (11) use logistic regression to differentiate between the Delta and Omicron variants. They delineate the two virus variants through the frequency of occurrence of the symptoms. For the analysis presented, symptom diary data covering a 2-year period and four prevailing dominant variants are available focusing on symptoms during the first 7 days.

## Materials and methods

In this section, we discuss the characteristics of the dataset, the specific data processing steps, and different model architectures, which overcome the challenges of the dataset with a focus on the interpretability of the model.

### Dataset

The dataset is provided by the health department in Cologne. It only includes index and contact persons with registered residence in cologne. Persons who only spent their quarantine in Cologne or came back from travels are not included in the dataset. It includes symptom profiles (SPs) of 90,244 patients with COVID-19 and 75,340 contact persons notified between March 2020 and February 2022. SPs contain information about the reported symptoms, self-assessed health, immunization status, and demographical data of the subjects. The SPs cover 46 and 42% of all notified cases in Cologne (index and contact persons) during the study period, respectively. Index persons include PCR-confirmed, antigen-positive persons, pending PCR confirmation persons identified by the laboratory, and self-reporting persons. Contact persons include individuals, who have had defined contact to an index person and are not infected with COVID-19 based on the criteria of Robert Koch Institute (RKI) for close contacts (12). SPs contain between 1 and 52 entries, resulting in a total number of 561,275 entries (236,544 entries of contact persons and 324,731 entries of index persons). Every diary entry includes information about the presence or absence of any symptom. In case of symptom occurrence, specific information is provided about the presence of one or more of the following symptoms: cough, dysgeusia and dysosmia, fatigue, running nose, fever, headache, sore throat, diarrhea, limb pain, nausea, and exanthema/skin rash. Further variables entail date of notification, date of symptom reporting, symptom onset, immunization status, age, and biological sex.

No systematic information is given regarding the virus variant for each SP. Therefore, we assign each entry a virus variant based on the prevailing dominant variants (PDV) in Cologne according to laboratory report analysis by the Virology Institute, University Cologne (unpublished data). The following four periods are defined by the data: (1) 02/2020–03/2021 Wildtype, (2) 04/2021–06/2021 Alpha, (3) 07/2021–26/12/2021 Delta, and (4) 27/12/2021–02/2022 Omicron. The variant of coronavirus that was detected in humans for the first time in 2019 is referred as Wildtype virus. Alpha, Delta, and Omicron are mutation of the Wildtype virus.

### Data processing

To obtain the training data for the classification of prevailing dominant virus variants (PDVs) and contact persons, several assumptions are made, and preprocessing steps are performed.

In a first step, the following exclusion criteria are applied:

SPs with a gap of 14 or more days between two consecutive entries, or an absolute difference of more than 7 days between the onset of symptoms and reporting day are excluded, to achieve a better alignment of the SPs.

Some assumptions are made regarding the immunization status because no vaccinations were available at the beginning of the study period during the predominance of the Wildtype variant. Therefore, the immunization status was introduced to the symptom diaries only after 8 February 2021 (opening date of the vaccination centers). Up to this date, we define the immunization status of all contact and index person as *no immunization*. After 8 February 2021, the vaccination status is systematically recorded and all SPs with unknown immunization status are excluded from the dataset.

The last exclusion criterion involves index and contact persons with an asymptomatic course. The goal of the classification task is to determine symptom patterns of the different prevailing dominant variants. Asymptomatic courses do not add any additional information to the dataset. Since asymptomatic courses can occur with any virus variant and do not differ, it is not possible to correctly assign asymptomatic courses to the individual variants.

The second step includes the preparation of the training and validation sets. The training dataset only includes SPs which have an entry for at least day 1 and day 7, whereby day 1 corresponds to the first symptom diary entry and is not aligned with the symptom onset of the person. The validation dataset only includes SPs which have an entry for at least day 1 and day 6. SPs contained in the training dataset are removed from the validation dataset, so that the training and validation dataset are disjoint.

**Figure 1:**
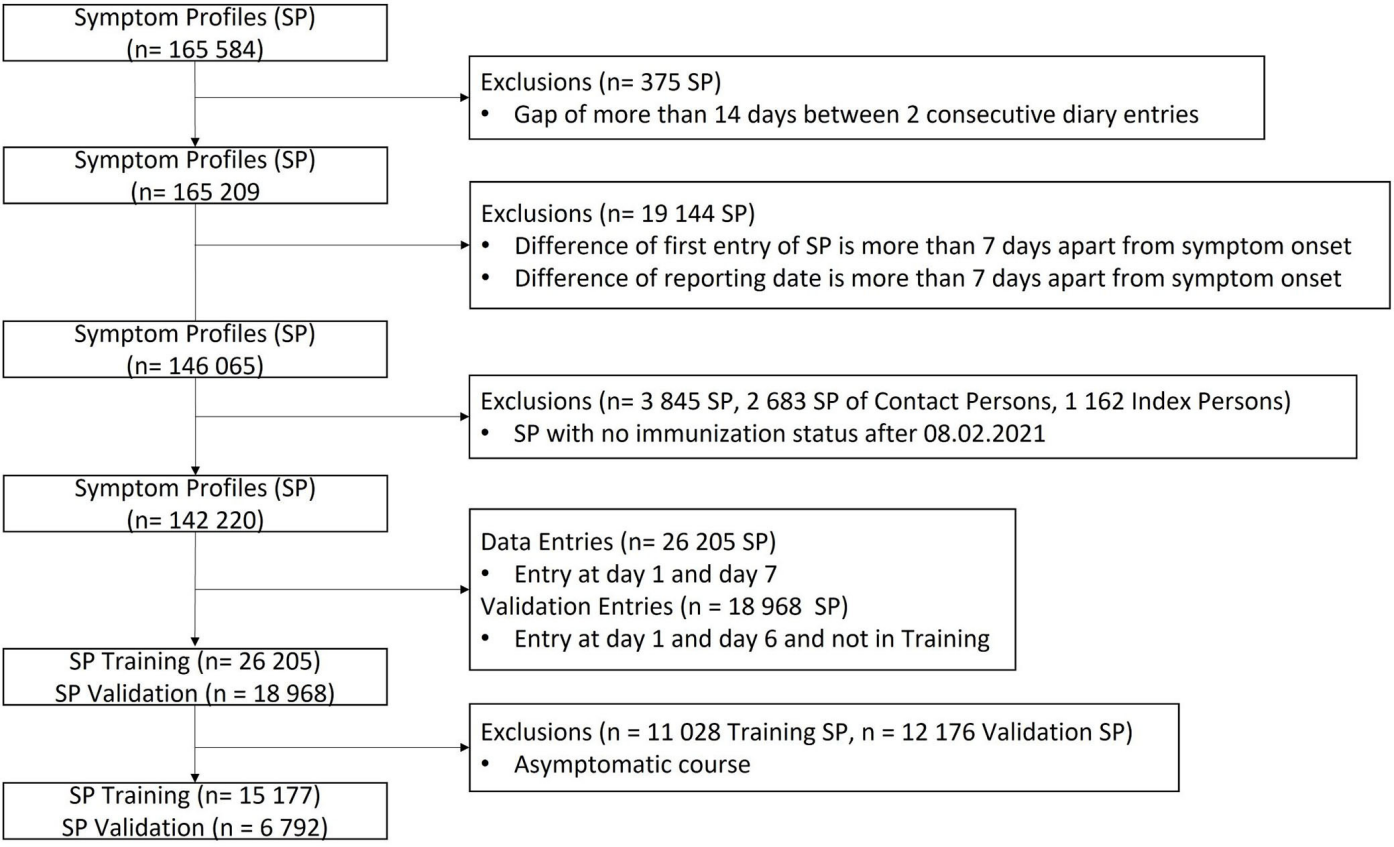
Data processing process, which includes the exclusion of unwanted SPs as well as the split into training and validation set.

The SPs of the validation and training set are restricted to the symptoms which occur during the first 6 (validation set) or 7 (training set) days. Symptoms, which appear after these days, are not considered in the training or validation data. Next to the symptoms, the SPs contain information about immunization status as well as age and biological sex. Missing values are filled with a dummy value−1. After all (pre)processing steps, the training set contains 15,177 and the validation set 6,792 SPs. The data processing process is visualized in Figure 1.

The number of SPs for the PDVs varies widely. This results in an imbalance between the different classes in the defined training and validation dataset (Table 1). The datasets contain mainly SPs from period 1 (PDV Wildtype, 44% of training set, 38% of validation set) and significant less from period 4 (PDV Omicron, 6% of training set, 9% of validation set). The number of SPs per class can differ by a factor of about 7. The different frequencies of PDVs are not related to the occurrence of positive COVID-19 cases in the assigned periods, but to the regular completion of symptom diaries. During periods of high incidence, such as Omicron, the Cologne health department did not have the capacity to monitor the completion of symptom diaries of all COVID-19-positive citizens. In addition, the orders for isolation duration in Germany changed in January 2022, and de-isolation was possible after 7 days instead of 10 days after symptom onset. Therefore, the SP of the index persons may not meet our criteria for inclusion in the datasets. This imbalance must be considered during the model selection as well as during the training, evaluation, and validation.

**Table 1:** Overview over the imbalanced distribution of prevailing dominant virus variant and contact persons in training, validation, and total dataset.

	**Contact persons**	**Index persons**			
**PDV at time of infection**		**Wildtype**	**Alpha**	**Delta**	**Omicron**
Training dataset	2,952	6,812	1,945	2,544	924
Validation dataset	1,322	2,588	898	1,395	589
Total	4,274	9,400	2,843	3,939	1,513

### Model description

For the classification of the PDVs and contact persons, three architectures with different characteristics regarding imbalanced datasets and interpretability are used: decision tree (DT), balanced random forest (BRF), and RUSBoostClassifier (RUS). Hyperparameters are tuned through cross-validation, and a final model is trained with the whole training dataset. The trained models are validated by the validation set. The exact training and validation procedure is described in Supplementary Data 1.

**Decision tree** DT is a tree-based classification algorithm. The most important advantages of this algorithm are the high interpretability (at least with few nodes) and the automatic selection of the most important features of the dataset. Furthermore, decision rules can be derived from the resulting tree through the paths from root to leaves. DTs also have the possibility of adapting the class weights in case of an imbalanced dataset.

**Balanced random forests** BRFs are random forests which are specialized on the problem of imbalanced datasets. To train the single DTs of the random forest, balanced bootstrap under sampling is used: All observations of the stronger represented class are removed from the training set of one DT equal often. As result, every observation of an overrepresented class is used equal often for the training of a DT. The trained DTs are combined to an ensemble and perform a majority voting for the prediction. Combining many DTs to an ensemble reduces the interpretability of the model.

**RUSboostclassifier** RUS is based on the AdaBoost Algorithm and adapted for the problem of imbalanced datasets. DT is used as a base estimator for RUS. During every boosting step (training of a base model), a random under sampling is performed. This means datapoints of the dominating class are not considered at random for the training to create a balanced dataset. Through the combination of many DTs, the architecture has a reduced interpretability.

For the evaluation and validation of the models, three different metrics are used: area under the receiver operating curve (AUC-ROC), sensitivity, and specificity. The AUC-ROC plots the true positive rate (TPR = sensitivity) against the false positive rate (FPR = 1- specificity) and is a curve of probabilities. Sensitivity is the probability of a sample being classified as positive, and its ground truth is positive, and specificity is the probability of a sample being classified as negative, and its ground truth is negative.

To decide which of the three models is used to perform the classification of the PDVs and contact persons, the complexity of the evaluation of the model is reduced to the binary classification problem: *index* vs. *contact persons*. Nevertheless, the model is trained on all classes; only for the evaluation and validation, all PDVs are combined to one class (index persons). The model with the highest sensitivity and specificity on training and validation set is chosen and used to evaluate and validate the specific classification problem: *all PDVs* vs. *contact persons*. If the metrics do not show clear results, the model with the highest interpretability and the simplest architecture is preferred.

## Results

In this section, we discuss and compare the results of the three models for the classification task. The evaluation of the models specifies how well the classification problem can be solved and the validation of the models determines how useful the models are, mainly with respect to unseen new data. The results are presented in subsections addressing the evaluation and validation of the two mentioned classification problems:

- The binary classification problem: *index* vs. *contact persons*.
- The more complex classification problem: *PDVs* vs. *contact persons*.

### Index vs. contact person evaluation

Sensitivity and specificity are used to compare the evaluation results of the three models (DT, BRF, and RUS) for the simplified binary classification problem: *index* vs. *contact persons*. All three models achieve nearly similar evaluation results (sensitivity [0.92–0.94], specificity [0.41–0.45], detailed information in Supplementary Table 1). The five features with the highest impact on the prediction are immunization, dysgeusia and dysosmia, cough, absence of all symptoms for at least 1 day, and limb pain, for DT; immunization, cough, dysgeusia and dysosmia, absence of all symptoms for at least 1 day, and running nose for BRF, and immunization, dysgeusia and dysosmia, cough, headache, and absence of all symptoms for at least 1 day for RUS. All three models focus on the attributes on immunization, dysgeusia and dysosmia, absence of all symptoms for at least 1 day, and cough.

### Index vs. contact person validation

The validation of *the index* vs. *contact person* classification problem reveals a sensitivity between 0.91 and 0.94 and a specificity between 0.37 and 0.45 (detailed results in Supplementary Table 1). The results on the validation set differ only slightly from the results of the training set (maximum difference in sensitivity: 0.01; maximum difference in specificity: 0.04).

**Figure 2:**
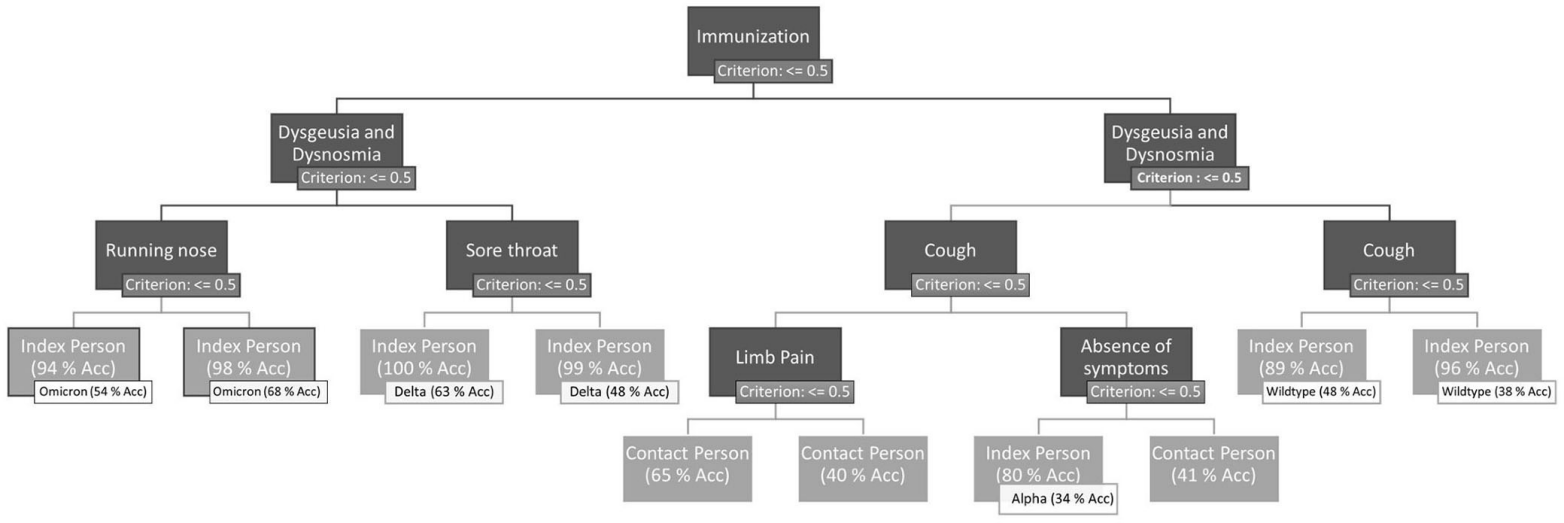
Final decision tree after hyperparameter optimization, trained on the whole training dataset. Leaves of the DT are evaluated (accuracy of the examples of the leaves) on the one hand for the binary classification problem index vs. contact person and on the other hand for the more complex problem prevailing dominant variants vs. contact persons. If a symptom is present, the right path of the node is chosen. The left path represents the absence of the symptom. Immunization is treated as a symptom. Therefore, immunization = 1 means that the person has no immunization by a previous infection or any vaccination.

As shown in Figure 2, DT uses the features of dysgeusia and dysosmia, absence of all symptoms for at least 1 day, runny nose, sore throat, limb pain, cough, and immunization status to distinguish between contact and index persons.

For further evaluation and validation, DT is used as a classification model. If we take a closer look at the decision tree, we can see that some paths result in the same class. This is partly because the tree has been trained on the complex problem *PDVs* vs. *contact persons* and partly because the presence or absence of a symptom can increase or decrease the probability of correct classification.

### PDVs vs. contact person evaluation

The AUC-ROC for the different classes of the DT for the classification problem *PDVs* vs. *contact persons* ranges from 0.60 AUC-ROC for Alpha to 0.89 AUC-ROC for Omicron. The detailed results can be found in Supplementary Table 2. An AUC-ROC of 0.89 allows to distinguish an Omicron index person from the remaining classes (other PDVs and contact persons) with a probability of 89%. Even with the complex classification problem, there are several paths that lead to the same virus variant (see Figure 2). Through the absence or presence of different symptoms, the probabilities of the nodes, which show the confidence of the virus variant classifications, can differ.

**Figure 3:**
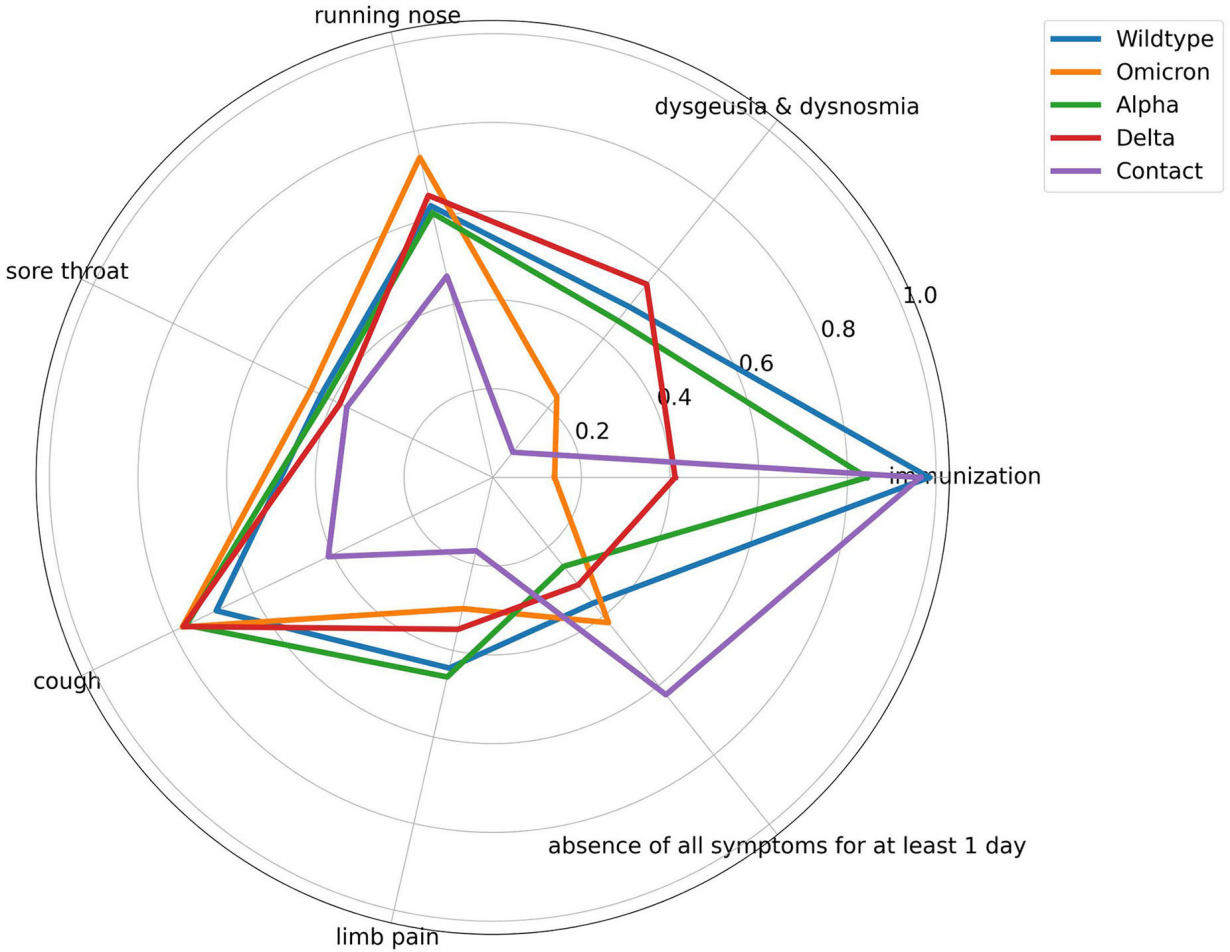
Spider plot of averaged symptom profile for the different prevailing dominant virus variants and contact persons. All symptoms range from [0,1] with 0 means symptom does not occur and 1 means symptom occurs. Immunization ranges from [0, 1], with 1 means no vaccination, and 0 means any type of vaccination regardless of the number.

**Figure 4:**
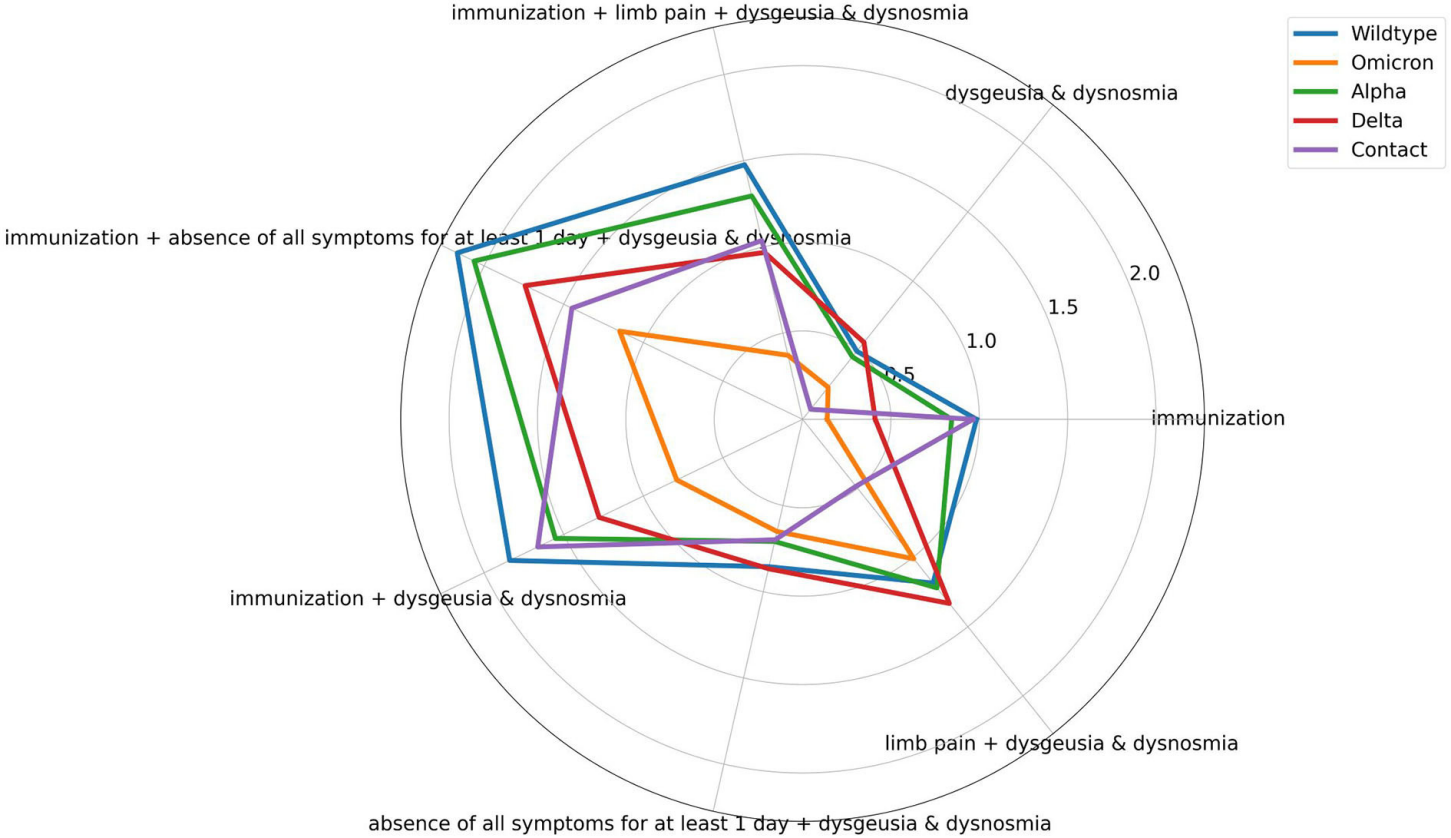
Spider plot of averaged symptom combinations for the different prevailing dominant virus variants and contact persons revealed from the decision tree. Symptoms can range from [0,1], whereby 0 represents the absence and 1 the occurrence of the symptom. Immunization can range from [0, 1], whereby 1 represents no immunization, and 0 means any type of vaccination regardless of the number.

To achieve a better impression of the difference between the symptoms of the PDVs and the corresponding classification performance, Figures 3, 4 visualize the average symptoms and average symptom combinations of the variants, revealed from the DT.

### PDVs vs. contact person validation

Using the 6-day benchmark set as validation, the AUC-ROC does not change significantly (AUC-ROC Alpha = 0.63; AUC-ROC Omicron: 0.87; maximum difference of 0.03 to evaluation results; detailed results in Supplementary Table 2). Alpha continues to achieve the poorest performance, Omicron the best.

## Discussion

During the SARS-CoV-2 pandemic in Germany, digital symptom diaries are developed and recommended for the use by infected people and their contact persons for the first time. A study of more than 165,000 anonymized diary entries of affected (index and contact persons) individuals within the municipality of the City of Cologne with around 1.1 million inhabitants is used. This innovative option allowed in a well-defined nominator and denominator population the use of the diaries for monitoring purposes, for example, deterioration of health status and the analysis of patterns of symptoms at population level over time both in patients and in contact persons and their potential use in a predictive model.

In the following, we first focus on the interpretation of the results of the two classification problems: *index* vs. *contact persons* and *PDVs* vs. *contact persons*. During the development of a prediction model, we consider the recommendation for transparent reporting of a multivariable prediction model as suggested in the TRIPOD statement by Collins et al. (16). Second, we relate the results to previous research, name limitations, and strengths of the study and provide an outlook for possible further research.

### Index vs. contact person

The three applied models (DT, BRF, and RUS) all achieve a sensitivity between 0.92 and 0.94 and a specificity between 0.41 and 0.45. A striking feature of all three models is the high difference between the sensitivity and specificity. Because the model is trained for the complex classification task *PDVs* vs. *contact persons* but evaluated for the simpler problem *index* vs. *contact persons*, all misclassifications between the different PDVs are not considered and are evaluated as true positives. As a result, the sensitivity of the simpler classification problem increases. Looking at the features the three models focus on, we can determine that immunization, cough, absence of all symptoms for at least 1 day, and dysgeusia and dysosmia contain the most information to differentiate between index and contact persons, because these features appear in all models.

By comparing the training and validation sets' evaluation metrics, we can see that the difference of the sensitivity and specificity is low (max difference of sensitivity: 0.03 and max difference of specificity: 0.04) for all three models. Only including unseen data in the validation set, we can conclude that the predictions of the model are useful. Furthermore, it shows the ability of generalization of the model and leads to the conclusion that none or a low amount of overfitting occurs during the training.

The additional evaluation and validation for the complex classification problem *PDVs* vs. *contact persons* are only done with the DT model. The decision is made based on the following: First, the model achieves slightly better results on the validation set than the other two. Second, DTs are the simplest and best interpretable model of the three. Third, a good visualization of the final model can be achieved with DTs. These characteristics are preferred due to good understanding and comprehensibility of the analyses.

### PDVs vs. contact person

The evaluation and validation of the DT for the complex classification problem *PDVs* vs. *contact persons* show significant difference in the performance of the classifications of the different PDVs and contact persons. The different variants achieve AUC-ROC from 0.6 to 0.89. To reveal the reasons for the different recognition performances, the influence of the single symptoms for each class is visually analyzed by a spider plot (Figure 3). The exact average occurrence of the symptoms in the variants can be seen in Supplementary Table 3. The symptoms have different impacts on the decision-making process, as can be seen through the different averaged symptoms for the different PDVs and contact persons. Sore throat, for example, has a low impact, and therefore, the average symptom does not differ much between the different PDVs. In contrast, dysgeusia and dysosmia have a larger difference and have a higher impact on the prediction. Nevertheless, Wildtype and Alpha have very similar averaged symptom profiles. This can explain the poorer classification performance of the DT. The DT does not decide on the value on only one symptom, but on symptom combinations. Figure 4 shows averaged symptom combinations, revealed from the DT (paths from root to leaves), for the different PDVs and contact persons. Using symptom combinations instead of single symptoms, the difference in the symptomatic of the PDVs and contact persons is easier to recognize. Same as in Figure 3, Wildtype and Alpha have quite similar averaged symptom combinations, which hint to the poorer classification performance.

Comparing the results of the evaluation and validation of the complex classification model, the results show again only a small difference (max AUC-ROC difference: 0.03) in the performance, as in the simpler classification problem (index vs. contact persons). We can again conclude that the model achieves useful results.

### Strengths and weaknesses of this study

Digital symptom diaries are a powerful tool to observe patients with COVID-19 in isolation (2, 5). They primarily allow individual monitoring and risk stratification for follow-up about the development of the course in terms of improvement and worsening of symptoms. Symptom diaries are a low-threshold tool that is easy to use at any time after login and can be operated on PC or smartphone. The acceptance rate of using the digital diary for the communication with the health department is around 35% (health department City of Cologne, unpublished data). It can be assumed that in future, this rate may increase since more people get used to the tool. Furthermore, the infections of the population of vulnerable people such as people in nursing homes or in hospitals are not recorded by digital symptom diaries, but by the nursing stuff.

Machine learning supports index person identification by symptom analysis and thus can become a valuable tool alongside specific laboratory diagnostics. As these analyses show, attention to symptoms should be a high priority in clinical practice and should not be abandoned in favor of laboratory diagnostics. Using symptoms for the identification of infected persons can offer great benefit if area-wide testing facilities are not available. It could allow a targeted assignment to testing opportunities. The model primarily allows retrospective assignment of symptom patterns and hence is in the current mode not suitable for early diagnosis; in contrast, PCR diagnostics is possible after contact even before symptom onset. However, infections are not exclusively detected by PCR testing, and sequencing is not performed for every sample. The viral variant often remains undetermined consequently. As the variants cause different severities of the disease course of COVID-19, the model can be used to predict the variant of individuals as well as a change in the dominant virus variant in the observed population. In case of a variant with a severe course, early and more intense actions can be prompted.

One limitation of this study is the missing recording of the symptom's strength. The diary only provides the option of symptom presence or absence for every entry. A subjective estimation of the severity of symptoms provides additional information to specify the SP of different PDVs. Different PDVs have similar symptoms, but the symptoms can occur with different severities. This grading of the symptoms could help the ML algorithms to differentiate more precisely between the classes. However, the individual assessment of the symptoms may be difficult for users and requires more time. Therefore, the query needs to be done as simple as possible.

Nevertheless, despite the missing strength of the symptoms and the subjective assessment of the symptoms of the patients, we can distinguish contact and index persons, as well as the different PDVs among each other. To our best knowledge, we developed the first model, which distinguishes more than two different variants of the virus only by using symptoms of the patients. So far, only gene sequences and CT + X-rays, or RNA strands and linguistic methods (13) are used. Through logistic regression, only Delta and Omicron are distinguished through symptom diaries (11).

### Potentials for public health services and outlook

To go one step further, in addition to distinguishing index and contact persons, as well as PDVs, the recognition of new variants can be of interest. For this purpose, one-class classification algorithms (14) can be used. The model is trained on all known PDVs. If a new PDV with different symptom pattern occurs, the model will classify it as an outlier. New mutations of the virus can be recognized, and in a period of transition between two variants, the model can determine the variant of infected persons.

The model can be permanently integrated in the digital symptom diary software (DiKoMa) of the City of Cologne. When a particular constellation of symptoms occurs, the software could suggest a test or, if laboratory capacity is limited or unavailable, help decide whether isolation is recommended; it could also be used to detect new symptom patterns, which could then lead to a search for new virus variants (15). This is also of great importance in settings where sequencing of specimen material cannot be easily performed and therefore assignment to variants with different clinical disease severity is otherwise not possible.

The results of the model can also be used for public health messaging, for example, to inform the public about the high likelihood for an infection once a certain symptom constellation occurs. By identifying index persons at the earliest possible stage, the spread of the virus can be counteracted more efficiently by identifying further contact persons of this index.

The evaluation and assignment of the symptom load in the population can thus enable monitoring of epidemics and pandemics independent of individual testing.

## Data availability statement

The data presented in this study is not readily available, as it belongs to the health authority and cannot be published, under German infection law. Further inquiries can be directed to the corresponding author/s.

## Ethics statement

The studies involving human participants were reviewed and approved by the data was collected due to the German infection protection act, so there was no need to get individual agreement. All sensitve data was stored on a secured server inside the city network and all database procedures were supervised and stated by the data protection responsible person oft the city on the base of German data protecion act. There was a contract which regulated the data handling between the parties who worked with the data and the data was anonymized before sent to the parties. Written informed consent from the participants' legal guardian/next of kin was not required to participate in this study in accordance with the national legislation and the institutional requirements.

## Author contributions

SK, BG, FN, SG, AW, MB, and AK designed the study. SK and SG did the data analysis. BG, AK-W, AK, AW, and MB collected data. SK, BG, FN, AK, AK-W, MB, and SR wrote and revised the manuscript. All authors contributed to the article and approved the submitted version.

## Conflict of interest

The authors declare that the research was conducted in the absence of any commercial or financial relationships that could be construed as a potential conflict of interest.

## Publisher's note

All claims expressed in this article are solely those of the authors and do not necessarily represent those of their affiliated organizations, or those of the publisher, the editors and the reviewers. Any product that may be evaluated in this article, or claim that may be made by its manufacturer, is not guaranteed or endorsed by the publisher.
