# Supplementary material for: Symptom diaries as a digital tool to detect SARS-CoV-2 infections and differentiate between prevalent variants
Source: Front Public Health. 2022 Nov 14;10:1030939. doi: 10.3389/fpubh.2022.1030939 (PMC9701827; doi:10.3389/fpubh.2022.1030939)
Supplement: Supplementary file 1 [file Data_Sheet_1.DOCX]

Supplementary Material

**Supplementary Data 1: Model Training and Validation Procedure**

After the training and validation sets are prepared, the best hyperparameters of the three models (Decision Tree, Balanced Random Forest, Random Under Sampling Boosting Classifier) are determined through a grid search with a stratified 5-fold cross validation. We are using a stratified K-fold-cross validation because the age and gender distribution over the prevailing dominant variants periods differ significantly. We choose the best hyperparameters based on the accuracy, precision and recall of the resulting models. For the evaluation, 10 times stratified 5-fold cross validation is performed with the chosen hyperparameters. The best hyperparameters are also used to train the final model on all training data. The final model is validated with the validation set. For the evaluation and validation of the model, we use Area Under the Receiver Operating Curve (ROC AUC) next to accuracy as metrics.

**Supplementary Data 2:** Questions asked and possible answers in the symptom diary

- Fever measured? Yes/No
- Fever? Yes/No
- Fever temperature in degrees Celsius?
- Sore throat? Yes/No
- Cough? No cough/dry cough/yellow-green mucus/bloody mucus
- Other symptoms? runny nose/headache/body aches/tiredness/diarrhea/disgeusia-dysnosmia/exanthema-rash
