# Supplementary material for: Symptom diaries as a digital tool to detect SARS-CoV-2 infections and differentiate between prevalent variants
Source: Front Public Health. 2022 Nov 14;10:1030939. doi: 10.3389/fpubh.2022.1030939 (PMC9701827; doi:10.3389/fpubh.2022.1030939)
Supplement: Supplementary file 2 [file Table_1.docx]

Supplementary Material

Supplementary Table 1: Detailed results of decision tree (DT), balanced random forest (BRF) and random under sampling boost classifier (RUS) for the classification problem contact vs index persons for the training (10-Fold-Cross Validation) and validation (6-day Benchmark) dataset.

| Result and Benchmark Table | | 10-Fold-Cross Validation | | 6-day Benchmark | |
| --- | --- | --- | --- | --- | --- |
|  |  | Sensitivity | Specificity | Sensitivity | Specificity |
| Contact vs Index | DT | 0.92 | 0.45 | 0.91 | 0.45 |
|  | BRF | 0.94 | 0.41 | 0.94 | 0.37 |
|  | RUS | 0.94 | 0.45 | 0.91 | 0.44 |
