# Supplementary material for: Symptom diaries as a digital tool to detect SARS-CoV-2 infections and differentiate between prevalent variants
Source: Front Public Health. 2022 Nov 14;10:1030939. doi: 10.3389/fpubh.2022.1030939 (PMC9701827; doi:10.3389/fpubh.2022.1030939)
Supplement: Supplementary file 3 [file Table_2.docx]

Supplementary Material

Supplementary Table 2: Detailed results of decision tree for the classification problem prevalent dominant variants and contact persons: ROC-AUC for the specific prevalent dominant variants and contact persons for the training (10-Fold-Cross Validation) and validation (6-day Benchmark) dataset.

| Result and Benchmark Table | | 10-Fold-Cross Validation | 6-day Benchmark |
| --- | --- | --- | --- |
|  | | ROC-AUC | ROC-AUC |
| prevalent dominant variants detection using decision tree model | Contact | 0.83 | 0.80 |
|  | Wildtype | 0.75 | 0.75 |
|  | Alpha | 0.60 | 0.63 |
|  | Delta | 0.79 | 0.77 |
|  | Omicron | 0.89 | 0.87 |
