# Supplementary material for: Symptom diaries as a digital tool to detect SARS-CoV-2 infections and differentiate between prevalent variants
Source: Front Public Health. 2022 Nov 14;10:1030939. doi: 10.3389/fpubh.2022.1030939 (PMC9701827; doi:10.3389/fpubh.2022.1030939)
Supplement: Supplementary file 4 [file Table_3.docx]

Supplementary Material

**Supplementary Table 3** Mean presence of symptoms in cases with different prevalent dominant variants and contact persons. All symptoms range from [0,1] with 0 means symptom is not occurred and 1 means symptom occurs. Immunization ranges from [0, 1], with 1 means no vaccination, 0 means any type of vaccination regardless of the number.

|  | Contact | Wildtype | Alpha | Delta | Omicron |
| --- | --- | --- | --- | --- | --- |
| being asymptomatic | 0.63 | 0.36 | 0.26 | 0.31 | 0.42 |
| fever | 0.06 | 0.21 | 0.23 | 0.19 | 0.11 |
| sore throat | 0.37 | 0.43 | 0.42 | 0.38 | 0.45 |
| cough | 0.41 | 0.69 | 0.77 | 0.77 | 0.78 |
| running nose | 0.47 | 0.63 | 0.61 | 0.65 | 0.74 |
| fatigue | 0.37 | 0.64 | 0.64 | 0.56 | 0.58 |
| dysgeusia +dysnosmia | 0.07 | 0.49 | 0.45 | 0.56 | 0.23 |
| headache | 0.45 | 0.60 | 0.58 | 0.50 | 0.50 |
| nausea | 0.07 | 0.16 | 0.17 | 0.13 | 0.10 |
| limp pain | 0.17 | 0.44 | 0.46 | 0.35 | 0.30 |
| exanthema | 0.01 | 0.02 | 0.03 | 0.03 | 0.03 |
| diarrhea | 0.08 | 0.15 | 0.15 | 0.11 | 0.08 |
| immunization | 0.96 | 0.98 | 0.84 | 0.41 | 0.14 |
